# Rural healthcare providers coping with clinical care delivery challenges: lessons from three health centres in Ghana

**DOI:** 10.1186/s12875-021-01379-y

**Published:** 2021-02-05

**Authors:** Vitalis Bawontuo, Augustine Adomah-Afari, Williams W. Amoah, Desmond Kuupiel, Irene Akua Agyepong

**Affiliations:** 1 Faculty of Health and Allied Sciences, Catholic University College of Ghana, Fiapre, Ghana; 2 Research for Sustainable Development Consult (r4sd consult), Sunyani, Ghana; 3 School of Public Health, University of Ghana, Legon, Ghana; 4 Centre for Evidence-based Health Care, Division of Epidemiology and Biostatistics, Department of Global Health, Faculty of Medicine and Health Sciences, Stellenbosch University, Cape Town, South Africa; 5 Ghana Health Service, Accra, Ghana

**Keywords:** Rural healthcare providers, Borrowing, Knowledge sharing, Multi-tasking

## Abstract

**Background:**

Rural settings in low- and middle-income countries are bedeviled with poverty and high disease burden, and lack adequate resources to deliver quality healthcare to the population. Drug shortage and inadequate number and skill-mix of healthcare providers is very common in rural health facilities. Hence, rural healthcare providers have no choice but to be innovative and introduce some strategies to cope with health delivery challenges at the health centre levels. This study explored how and why rural healthcare providers cope with clinical care delivery challenges at the health centre levels in Ghana.

**Methods:**

This study was a multiple case studies involving three districts: Bongo, Kintampo North, and Juaboso districts. In each case study district, a cross-sectional design was used to explore the research question. Purposive sampling technique was used to select study sites and the study participants. The authors conducted 11 interviews, 9 focus group discussions (involving 61 participants), and 9-week participant observation (in 3 health centres). Transcription of the voice-recordings was done verbatim, cleaned and imported into the Nvivo version 11 platform for analysis. Data was analysed using the inductive content analysis approach. Ethical clearance was granted by the Ethics Review Committee of the Ghana Health Service.

**Results:**

The study found three main coping strategies (borrowing, knowledge sharing and multi-tasking). First, borrowing arrangements among primary health care institutions help to address the periodic shortage of medical supplies at the health centres. Secondly, knowledge sharing among healthcare providers mitigates skills gap during service delivery; and finally, rural healthcare providers use multi-tasking to avert staff inadequacy challenges during service delivery at the health centre levels.

**Conclusion:**

Borrowing, knowledge sharing, and multi-tasking are coping strategies that are sustaining and potentially improving health outcomes at the district levels in Ghana. We recommend that health facilities across all levels of care in Ghana and other settings with similar challenges could adopt and modify these strategies in order to ensure quality healthcare delivery amidst delivery challenges.

## Background

Health systems worldwide are ambitious to enhance performance by improving access, quality, equity and financial protection during health service delivery [25]. This desire supports the core values of the Primary Health Care (PHC) initiative under the Alma Ata declaration in 1978 [4, 24]. For this reason, efforts to strengthen health systems are directed towards improving the performance of PHC institutions in order to meet the PHC requirements [15]. In Sub-Saharan Africa, most countries integrated the PHC concept into decentralised units (district health system), serving as building blocks of national health systems [5, 24]. Thus, a well-integrated PHC-based district health system is made-up of healthcare institutions that are linked in harmonious relationships to provide appropriate treatment for common diseases and injuries, basic laboratory services, essential drugs and referral services, as well as preventive healthcare services [3]. These core activities ensure that quality, affordable and comprehensive healthcare services are delivered to sick individuals, families and communities especially in rural settings [18].

In low and middle income countries, PHC is the first point of healthcare, responsible for ensuring person-centred, comprehensive and coordinated healthcare to rural communities [21]. Provided at the personal level, primary care services concentrate on the medical and health management of a child, adult, or family when the patient first presents to the formal health system [9, 21]. District-level PHC services are provided by a range of health workers, who have received the requisite training both technically and socially and work together as a team to deliver the needed services. These health workers include physicians, nurses, physician assistants, midwives, other paramedical staff, auxiliaries and community workers where applicable [22].

Ghana’s PHC system is structured into three service delivery levels: district hospitals, health centres and Community-based Health Planning and Services (CHPS) [7]. Traditionally, the health centre has been the first point of contact between the formal healthcare system and the client in the management of clinical conditions [23]. It is designed to provide basic curative as well as preventive services for adults and children as well as reproductive health services. Most health centres are situated in rural settings and serve populations of approximately 20,000 in their jurisdiction. These delivery levels work in a network model where resource sharing is an integral part of service delivery. Available resources for sharing at the PHC levels may include; human resource knowledge, skills and expertise; drugs and consumables; equipment; and information. In this light, a study confirmed that resource sharing is highly practiced among many local health departments; and organisations could take advantage of resource sharing practices to meet their needs [23]. This practice is particularly important in the rural setting where health resources are limited.

Rural settings in low and middle income countries are bedeviled with poverty and high disease burden [14], and also lack adequate resources to deliver quality healthcare to the population. For instance, drug shortage is very common in rural health facilities [6, 13]. Additionally, the number and skill-mix of health professionals who provide clinical care at the health centre levels is inadequate [16, 19]. Hence, provision of clinical care at the health centre levels potentially faces numerous challenges as rural health professionals strive to provide quality care to the people. These rural health professionals have no choice but to be innovative and introduce some strategies to cope with the clinical care delivery challenges. This paper seeks to explore how and why rural healthcare providers cope with clinical care delivery challenges at the health centre levels in Ghana.

## Methods

### Study design

The study used multiple case studies involving three districts selected from the northern (Bongo district), middle (Kintampo North district) and southern (Juaboso district) belts of Ghana to analyse how and why rural healthcare providers cope with clinical care delivery challenges in resource-constrained settings. A “case” is defined as a complex functioning unit, investigated in its natural context with a multitude of methods [26]. In this study, the case is defined as a health district which has a district health directorate, a district hospital and a functioning health centre. Bongo district was used as a nucleus district, while Kintampo North and Juaboso districts served as triangulation districts [20]. Bongo-Soe health centre was selected in the Bongo District, New Longoro health centre in the Kintampo North Municipal and Bonsu-Nkwanta health centre in the Juaboso District. In each case study district, a cross-sectional design was used to explore the research question.

### Data collection

Data collection for this study took place between 2015 and 2016. The study used mixed qualitative methods (observation, interviews and FGDs) to collect data. Three-week participant observation was done in each health centre in order to study how and why healthcare providers cope with clinical care challenges. Eleven [11] in-depth interviews, involving 3 district directors, 3 medical superintendents, 3 sub-district leaders and 2 deputy regional directors in-charge of clinical care, were conducted. Finally, nine focus group discussions (FGD), 3 FGDs in each case study district, were conducted. In each district, FGDs were conducted among selected district health managers, hospital frontline providers and health centre healthcare providers.

### Sampling

Purposive sampling was used to select health managers as interview participants and frontline healthcare providers as FGD participants. Thus, the study participants constituted managers of the district health institutions and their immediate supervisors, as well as the frontline healthcare providers of the district health services at the time of the study. A summary of the study participants in all three districts is presented in Table 1.

**Table 1:** Master table

**Health hazards**	**Superordinate themes**	**Constituent sub-themes**
	Stress	Anxiety
		Inadequate number of midwives
	Infection	Blood and liquor as sources
	Slip and fall	Splashes of liquor
	Assault	Poor labour outcomes
	Irritation and Burns	Use of corrosive chemicals/drugs

### Data analysis

Interviews and FGDs were voice-recorded and transcribed verbatim. The transcripts of the voice-recordings and field notes were cleaned and imported into the Nvivo (version 11) platform. The analysis was done using the inductive content analysis approach by coding to identify common patterns and inconsistencies. Common patterns in the data set were categorised into three main themes: borrowing, knowledge sharing and multi-tasking. The data was analysed further to understand how and why these themes are sustained.

## Results

The findings revealed that rural healthcare providers adopt three main coping strategies (borrowing, knowledge sharing and multi-tasking) in order to mitigate clinical care delivery challenges at the health centre levels in Ghana. These have been presented below.

### Borrowing of supplies and logistics

The analysis revealed that all three health centres borrow supplies and logistics from each other to address the challenges associated with inadequate or lack of medical supplies in order to facilitate health centre clinical care delivery. The borrowing initiative means that health centres, which lack medical supplies could approach the district hospital or other health centres to borrow the needed items and return same to the lender at a later date. Commonly used medical supplies to provide clinical care include; medicines, cotton, gauze, basic clinical and nursing equipment, laboratory equipment and reagents, patient folders, and detergents. However, across the three health centres, the study observed, and participants also reported that these essential medical supplies were inadequate, and some completely lacking, at the time the study was conducted:

[…] *Sometimes, we (health centres) run short of drugs, we cannot get common paracetamol for our clients* […] (IDI_P02_Region B).

An analysis of why the periodic shortage of medical supplies revealed that health centres are faced with cash flow challenges. Probing further the study found that, in the era of lack of government subvention to facilities, health centres rely on their Internally Generated Funds (IGF) to restock medicines and other resources for effective service delivery.

[…] B*udgetary allocations to the BMCs have dwindled or stopped completely* […] *Government is now funding our budgets through the services we provide* […] *So we just have to generate more money to survive* […] (FGD_DHFP_P04_Region C).

However, health centre IGF is affected by irregular payment schedules from the National Health Insurance Scheme (NHIS). Thus, this affects the availability of health resources at the health centre levels:

[…] *For drugs, we have a problem, because of NHIS* […] *For instance, they owe us about eight months, there are a lot of things that are short in the health centre* […] (IDI_P04_Region C).

Analysing how heath centres cope with these challenges, the study found that a borrowing **initiative** was introduced. In this instance, district hospitals allow health centres which run out of medical supplies to borrow from the hospital and replace at a later date:

[…] *With medicines and medical supplies, when they (health centres) need them, they come and we (hospital) give to them; and when they get them, they bring back to us* […] (FGD_DHM_P01_Region C).

Analysis of how the borrowing initiative works revealed both formal and informal borrowing mechanisms. Formal borrowing of medical supplies means that the borrowing arrangements are channeled through the hospital management for approval. For example:

[…] *If for any reason, sub-districts run short of medicines, folders or some consumables, they come and borrow (laughing)* […] *They call me (medical superintendent) and I give the items to them, and they replace these items later* […] (IDI_P03_Region C).

On the other hand, informal borrowing implies that district hospital transfers medical supplies to the health centres without the approval of hospital management, For instance, it was reported that healthcare providers at the district hospitals encouraged their colleagues at the health centre levels to collect items for use whenever they run out, and to replace them later:

[…] *Once the whole district ran out of folders, and we (hospital) had some folders in stock, so we called the sub-district to come and pick some folders, so that they can replace later* […] (FGD_DHFP_P06_Region C).

It was also reported that healthcare providers of health centres talk to their colleagues at the district hospitals to collect medical supplies and replace them later:

[…] *Some of the sub-districts just come straight to us (hospital frontline providers) to borrow, and depending on the item, we give and they return it later* […] (FGD_DHFP_P02_Region A).

Nonetheless, health centres also borrow from colleague health centres in the district. In this case, health centres contact and borrow from each other to beef-up shortage in medical supplies:

[…] *Yes, we borrow from other health centres, when we need something, we just call other health centres and if they have, we will borrow and give back to them when we get ours* […] (IDI_P04_Region C)

The analysis also revealed that medicines and medical supplies that are near expiry at the health centres could be swapped with new medicines and medical supplies at the district hospitals:

[…] *When sub-district drugs are about to expire, they bring the drugs to the hospital and they exchange for us* […] (IDI_P01_Region A).

Analysis of how and why this borrowing initiative is sustained revealed that health centre care providers are honest men and women. Thus, the borrowing arrangement between these PHC level institutions is sustained by trust. Participants responded to a question; ‘are there times they do not return borrowed items?’:

[…] *We are honest men and women (laughing)* […] (IDI_P03_Region C).

This coping strategy adopted by the healthcare providers has the potential to minimise waste, and as well ensure that quality drugs are available to support service delivery at the health centre levels. This effort could, therefore, improve health outcomes in the rural settings where resources are contrained.

### Knowledge sharing

Across the three districts, it was widely reported that health centres used knowledge sharing to manage healthcare providers’ skills gap during service delivery. In this study, knowledge sharing means healthcare providers at the health centre levels seek professional advice from healthcare providers at the district hospitals during service delivery. It was also observed that during clinical case management, healthcare providers at the health centres called on the medical superintendents or other colleagues who work at the hospitals for additional information in order to provide the needed care to the patients:

[…] *Whenever we (health centre staff) call, what we do not know they (district hospital staff) tell us and we continue with the treatment* […] (FGD_HCFP_P02_Region C).

The analysis confirmed that health centre healthcare providers constantly call the district hospital healthcare providers to discuss and seek case management guidance:

[…] *Whenever they have any problem, they call me (medical superintendent), we discuss the case and I assist them to manage the case; if they need to bring the patient here, I assist them to do that* […] (IDI_P02_Region A).

The study found that the knowledge sharing initiative have the potential to improve health outcomes in the district. Once knowledge is shared about a particular medical case, chances are that health centre healthcare providers would be able manage the case at the health centre level. Nonetheless, if the patient is referred to the hospital, the patient information is already known at the hospital level; and this facilitates continuity of care at the hospital:

[…] *At times, they also call for guidance in managing cases at the health centre level… If the directions given do not work, then they refer* […] *This is helping a lot to reduce our maternal deaths* […] (FGD_DHFP_P03_Region B).

Analysis of how knowledge sharing is sustained revealed that knowledge sharing has been institutionalized i.e. district hospital management officially informed health centres to call for professional advice whenever they face service delivery challenges:

[…] *Doctor said whenever we have a case, we have to call first; either they give us directives or they ask us to bring the case, so we share ideas* […] (FGD_HCFP_P01_Region C).

Further analysis of why knowledge sharing exists between district hospitals and health centres revealed that health centres recognise the expertise of the district hospitals and constantly engage them on how to manage cases at the health centre levels.

[…] *so they (health centres) see us (district hospitals) as their superiors and ask for support when they need it* […] (IDI_P03_Region C)

This initiative suggests that health centres seeking professional advice from the district hospitals has the potential to bridge knowledge gap in health centre service delivery.

### Multitasking

The study observed that health centre healthcare providers used multi-tasking to minimize staff inadequacy challenges during service delivery. In this study, multitasking means healthcare providers performing more than one task during service delivery. This approach allows healthcare providers to offer support to units within the health centre that have staffing challenges, and thus mitigate the effects of staff inadequacy during service delivery. It was revealed that health centres operate in a flexible environment where healthcare providers move from their assigned duty-post and voluntarily support over-burdened units during service delivery at the health centre.

First, the analysis revealed that health centre laboratories benefit from multitasking. Even though laboratory practice is technical, it was observed, and participants reported that working colleagues supported the laboratory services with task that did not require technical knowledge:

[…] *The laboratory receives a lot of support from colleagues in the health centre* […] *When the place is crowded, some colleagues come to help me with the registration of patients and entry of results, while I run the tests* […] (FGD_HCFP_P04_Region C).

Secondly, the analysis revealed that health centre dispensaries also benefit from multitasking. In all the three health centres, it was observed, and participants reported that the dispensaries were manned by one person who had multiple roles in the health centre. Hence, the dispensary sections of health centres were over-burdened and required support. In such situations, colleagues from other sections of the health centre move to support in dispensing drugs to patients:

[…] *At times, some people have to come in and help, because I am alone at the dispensary* […] (FGD_HCFP_P05_Region C).

Thirdly, health centre records sections also benefit from multitasking. The study revealed that these sections lack qualified personnel, and this translates into a service delivery burden, especially when outpatient attendance is high. In such instances, a number of healthcare providers gather at the records section to help retrieve patient folders before they return to their official duty points:

[…] *So people leave their places to support in retrieving folders, this morning, I came and saw you and the medical assistant retrieving folders (some laughing)* […] (FGD_HCFP_P06_Region C).

Finally, the analysis also revealed that health centre healthcare providers support skilled delivery services at the maternity section. A single midwife working in the health centre suggests that the maternity section requires support in order to conduct quality skilled delivery. Midwives at the health centre level workday and night to meet maternal healthcare needs of the rural communities. The health centre midwives provide antenatal and postnatal care, as well as conduct deliveries for pregnant women. During delivery, health centre midwives require support and colleague healthcare providers readily provide some form of support:

[…] *Anytime I call on anybody to support me at the maternity, they are always willing* […] (FGD_HCFP_P07_Region C).

Analysis of why multitasking is used during service delivery revealed that healthcare providers identify and prioritize sections of the health centre that require support in order to enhance quality of care. For instance, the records section is the starting point of the healthcare delivery process at the health centre. Therefore, any delays in this section affect the entire service delivery leading to long waiting times and patient dissatisfaction. Against this background, healthcare providers move to support the records sections of the health centres:

[…] *When patients delay at the records section, it affects the entire process* […] (IDI_P04_Region C)

Analysis of how multitasking is sustained revealed that there is high team spirit and love for the job among healthcare providers in the health centres. It was observed and participants report that healthcare providers are very hard working and remain committed to supporting each other in order to strengthen service delivery. Above all, the healthcare providers at the health centre love and respect each other:

[…] *We love each other, and we love the work* […] *As if they have selected us together, we are just okay with each other* […] *We are all hardworking and ready to support one another each other* […] (FGD_HCFP_P07_Region C).

This suggests that team-spirit among health centre healthcare providers has the potential to facilitate multitask approach to health service delivery. This effort is likely to improve health outcomes in the sub-districts, districts and the national level as a whole.

## Discussions

### Borrowing of supplies and logistics

The study found that shortage of medical supplies is one of the clinical care delivery challenges at the health centre levels in Ghana. This means that managers as well as service providers must be proactive to address these inevitable challenges in order to provide quality primary healthcare - providing health services in a manner that is consistent with the health-related needs of the population. In response, the healthcare providers have developed some coping mechanisms to ameliorate the periodic shortage of medical supplies. The study found “borrowing” to be a significant and reliable strategy to avert the occasional shortage of medical supplies in the district health system. Similarly, in a study to explore the responses of frontline healthcare workers to stock-outs of essentials medicines and equipment, the authors argued that healthcare workers access these medical supplies from other facilities [10].

Both “vertical borrowing” and “horizontal borrowing” were resorted to, in order to ensure continuity of healthcare delivery to rural populations. In this case, vertical borrowing is the situation whereby health centres borrow from district hospitals, which is a higher-level facility in the district health system. On the other hand, horizontal borrowing is when health centres borrow from their colleague health centres in other sub-districts within the district. As the findings revealed, district hospitals were a dependable lender to health centres when they needed medical supplies that were either unavailable at the medical stores (district/regional) or when there was lack of funds to acquire them. This finding corroborates a study, which emphasised the role of district hospitals in the efficient management of PHC services [17]. Likewise, convenience and speed may be among the benefits of the relationship in the horizontal borrowings that happen between sub-district level health facilities. This interest-free borrowing arrangement is worth commending since it serves as a better option to securing a loan from the banks to cater for such needs. Additionally, this interest-free arrangement is innovative since money is the major challenge facing health centres rather than mere shortage of supplies at the medical stores.

As the study revealed, some of the borrowing processes were formalised such that the request or arrangement must be in writing and channeled through the district hospital management. In contrast, the informal borrowing arrangement was based on individual health workers’ relationships and did not go through any appropriate documentation compared with the formal. This finding was consistent with a study, which argued that stock-outs were only reported when informal methods of stock-sharing did not secure top-up supplies [10]. This informal process could be a remedy for the bureaucratic processes associated with the formal process, thus making medical supplies timely available for use at the health centres.

However, there is the need to express genuine concern about the possibility of undesirable issues of near corruption emerging as the process is done at the blind side of management. Thus, this situation is likely to further weaken the health system and deny it of essential and already scarce resources. In as much as borrowing had served a good purpose and bridged the medical supplies gap created by periodic shortages, the district health system would be better-off if such arrangements were often formalised. Similarly, Hodes and colleagues indicated that informal borrowing of medical supplies had implications for understanding the frequency and severity of stock-outs, and for taking action to prevent and manage stock-outs effectively [10].

It is also noteworthy, that the study found trust to be the basis of the borrowing arrangements. The entities involved expected that there would be smooth replacement once the borrower secured the medical supplies they borrowed. This creates the impression that previous transactions and arrangements had been faithfully honoured and therefore, sustains future borrowing arrangements between district hospitals and health centres. This finding was consistent with a study, which argued that trust underpins the co-operation within health systems [8].

In a more fascinating revelation, it was found that in a situation where medical supplies at the health centres were nearing expiration, the health centres made arrangements with the district hospital and exchanged for newer ones, which were not near expiry. This arrangement was backed by the good reasoning that medical supplies consumption at the district hospital is huge and as a result, stocks move faster than at the health centres. As the study noted, medicines were the commonest medical supplies that went through this process. This is in every sense another innovative strategy that is worth emulating. It helps to prevent waste at the health centres. However, it must be considered as an important factor when planning or in the purchasing and supply chain management of both the district hospitals and health centres. This conforms to a study, which concluded that knowledge of medical supplies and equipment for PHC was a useful resource for those at national and district levels responsible for health planning and management, training, and managing medical stores [12].

### Knowledge sharing

Knowledge sharing is a top-down approach adopted to support service delivery at the sub-district and community levels. In this knowledge sharing approach, health centre healthcare providers call on the medical superintendent or other district hospital healthcare providers to seek professional advice in the course of managing cases at the health centres. This helps to bridge knowledge gap at the health centre level and also improve or facilitate quality care delivery to the rural population. Again, this approach is considered worthwhile because it saves time and resources. For instance, if the service or treatment being offered needs higher professional advice or input, in the absence of this knowledge sharing, the patient would have to be transferred to the district hospital and this comes with costs (e.g. transportation cost). Thus, this initiative is laudable in addressing patient referral challenges such as lack of ambulance or vehicle to safely transfer patients to the district hospital, as well as inadequate healthcare providers at the health centre level, which determines whether qualified staff accompanies referred patients to the district hospital. A study explored how healthcare professionals shared knowledge in the Ghanaian healthcare sector, and found that the healthcare facilities studied did not have any formal knowledge management systems, and therefore, healthcare professionals relied on informal conversations and seminars to share knowledge [1].

Another advantage of the knowledge sharing is that, it helps the providers at the health centres gain additional knowledge that would improve future service delivery. The findings revealed that knowledge sharing was a formal arrangement that the district health system had instituted between the district hospital and the health centres. This finding corroborates with a study, which concluded that knowledge sharing practices in healthcare delivery were strongly influenced by institutional structures [11]. Of course, this is to ensure positive impact of healthcare delivery on the district population health outcomes. As a participant concluded, the knowledge sharing has ‘*contributed to a decrease in the maternal death’* (IDI_P02_Region C) that they recorded.

### Multitasking

Multitasking emerged as a coping strategy among the health centre healthcare providers. The inadequate staff numbers at the health centre level means that healthcare providers need to support each other outside their assigned units or departments. For instance, the study revealed that, the records, dispensary, maternity and laboratory units always received support from staff of other units in the health centre. This practice was adopted to enhance the speed of service delivery and reduce waiting time. The records unit is usually crowded in the morning as many patients seeking treatment retrieve or register for a folder. To facilitate patients’ records retrieval for previous/returning visitors/patients and also register new patients, other healthcare providers who were available from other units joined the records officer to execute their duty/duties. This was not only peculiar to the records unit though. Multi-tasking was done in the spirit of teamwork, and the love and passion for their jobs because it was voluntary rather than a formalised role. In effect, the support reduces waiting time, which has been identified as a significant predictor of patients’ satisfaction [2].

As a limitation to the study, the study selected three districts out of 216 districts in order to explore how and why rural healthcare providers cope with clinical care delivery challenges at the health centre levels in Ghana. In this regard, the findings of the study based on these districts cannot be generalised to all districts in Ghana. In order to overcome this weakness, the study selected a district from each of the ecological zones in Ghana; Bongo District in the Upper East Region (Northern belt), Kintampo North in the Brong Ahafo Region (middle belt) and Juaboso District in the Western Region (southern belt). In this way, constructs / themes generated in this study were confirmed across the three districts, giving a diagonal representation of the study phenomenon.

## Conclusion

The findings of this study suggest that rural healthcare providers adopt various innovative and coping strategies to mitigate health delivery challenges at the health Centre levels in Ghana. Borrowing supplies and logistics, knowledge sharing, and multitasking are coping strategies that are sustainable and would potentially improve health outcomes in the district. The findings of this study also suggest that health facilities across all levels of care in Ghana and elsewhere with similar challenges could adopt and modify these strategies in order to ensure quality healthcare amidst delivery challenges.

## Data Availability

All essential data are within the manuscript. The transcripts are also available upon a request to Dr. Vitalis Bawontuo via bawontuovitalis@yahoo.com.
